# Mitral Valve Restenosis after Percutaneous Transmitral Valvuloplasty, Role of Continuous Inflammation

**DOI:** 10.15171/jcvtr.2014.010

**Published:** 2014-09-30

**Authors:** Mohammadali Ostovan, Amir Aslani, Shahima Abounajmi, Vida Razazi

**Affiliations:** ^1^Department of Cardiology, School of Medicine, Shiraz University of Medical Sciences, Shiraz, Iran; ^2^School of Management and Information, Shiraz University of Medical Sciences, Shiraz, Iran

**Keywords:** C-Reactive Protein (CRP), Inflammation, PTMC

## Abstract

*Introduction:* High sensitive C-Reactive Protein (hs-CRP) is increased in acute and chronic rheumatic fever (RF), but is unknown whether serum levels of hs-CRP is correlated with late restenosis of mitral valve (MV) after Percutaneous transvenous mitral commissurotomy (PTMC). The aim of this study is to determine relationship between hs-CRP and MV restenosis 48-36 months after performing PTMC.

*Methods:* A total of 50 patients who had undergone PTMC due to rheumatic etiology (41 female, 9 male; mean age 46 ± 11, range 27-71), all followed up on an out patients basis 36 months after PTMC, were included in the study. The hs-CRP was measured using an enzyme-linked immunosorbent assay (ELISA) kits.

*Results:* No association was found between hs-CRP level and mean transmitral valve gradient 36 months after PTMC, MV area by planimetry, pulmonary artery systolic pressure, mitral regurgitation grade, left atrial diameter, atrial fibrillation (AF) rhythm and Wilkins score.

*Conclusion:* Our study have shown that there is no association between hs-CRP and MV restenosis in patients with rheumatic heart disease (RHD) who underwent PTMC. Therefore, it has been postulated that inflammation is not a cause of post PTMC restenosis.

## Introduction

Chronic rheumatic mitral stenosis (CRMS) is a late sequel of rheumatic fever (RF), leading to rheumatic valve heart disease.^1^ Rheumatic valve heart disease is more common in females, sometimes up to twice as frequently as males.^1^ After an episode of RF, the latency period is 10-20 years or more before the onset of symptoms.^2^ In acute RF, there is inflammation and edema of the leaflets, leading to characteristic valve deformity. It has been considered that chronic rheumatic valve disease is usually the result of repeated episode of carditis alternating with healing and is characterized by the deposition of fibrous tissue.^3^ The pathogenic mechanism involved in the disease is autoimmune due to “antigen mimicry” between M protein epitopes of group A streptococci and heart protein which leads to valvular damage in susceptible patients.^4^ In developing countries, CRMS progresses more rapidly, presumably due to either a more sever rheumatic injury or repeated episodes of carditis due to streptococcal infections.^5^ Percutaneous transvenous mitral commissurotomy (PTMC) is a mainstay of the management of rheumatic mitral stenosis . PTMC, first described in 1984 by Inoue et al, has become a very popular technique over time, for selected patients with moderate to severe mitral stenosis (MS).^6^ High sensitive C-Reactive Protein (hs-CRP), a sensitive marker for systemic inflammation, is one of the acute phase protein and predicts morbidity and mortality in various clinical conditions.^7-11^ CRP assay provides useful information for the diagnosis, therapy and monitoring of inflammatory processes and associated diseases.^12-14^ Hs-CRP is increased in acute and chronic RF, but is unknown whether serum levels of hs-CRP is correlated with late restenosis of mitral valve (MV) after PTMC. If a role for continuous inflammation in restenosis after PTMC is determined, then anti-inflammatory measures may be helpful in prevention of restenosis .The aim of this study is to determine relationship between hs-CRP and MV restenosis 36-48 months after performing PTMC.

## Materials and methods

### Study population

A total of 50 patients who had undergone PTMC due to rheumatic etiology (41 female, 9 male; mean age 46 ± 11, range 27-71 ), all followed up on an out patients basis 36 months after PTMC, were included in the study. The number of female patients is more than males because the prevalence of rheumatic heart disease (RHD) in females is more than males. All patients were selected from Namazi, Shahid-Faghihi and Kowsar hospitals, Shiraz, since January 2011 till January 2012. We excluded patients with a history of infection, malignancy, acute and chronic inflammatory disorders, acute phase of Guillain -Barre syndrome and multiple sclerosis, a history of recent surgery or trauma, a history of acute myocardial infarction and coronary artery disease, as well as those with a history of statin consumption.

### Procedure

PTMC was done with a transvenous transseptal access to the left atrium and an Inoue balloon catheter that was inflated it within orifice under local anesthesia. A balloon size was calculated by below equation:

Balloon size (mm) = [Height ^(cm)^ / 10] + 10

The mean transmitral gradient was reducted to ≤5 mm Hg and mitral valve area (MVA) was increased to >1.5 cm^2^ or ≥50% of pre-procedural MVA.

### Transthoracic echocardiography

Two-dimentional and Doppler echocardiography were performed using a GE-Vivid Five System with a 2.5 MHz probe. Two-dimentional M-mode measurements were taken according to American Society of Echocardiography guidelines. The MVA was measured using the pressure half time method (PHT) and planimetry of the MV orifice from the short axis view at the tip of the mitral leaflets. The mean transmitral diastolic pressure gradient were determined using the Doppler method before and after PTMC and after 36 months. Pulmonary arterial systolic pressure (PASP) was calculated by measuring the velocity of the tricuspid regurgitation jet. Left atrial dimension and grade of mitral regurgitation (MR) were determined. MV morphology was calculated on the basis of the Wilkins scoring system included leaflet calcification, mobility and thickness and subvalvular apparatus degeneration.

### Hs-CRP serum level measurement

Blood samples were collected in small tubes (1.5 ml) using standard venipuncture techniques. Specimens were stored at –70^◦^C after centrifugation. The hs-CRP was measured using an enzyme-linked immunosorbent assay (ELISA) kits. Hs-CRP serum level was collected 36 months after PTMC.

### Statistical analysis

Data were analysed using the SPSS 16.0 package and were expressed as mean ± SD. Difference between mean values were tested by student’s *t*-test. Variate comparisons between groups were made with: Kruskal-Wallis test for multigroup comparisons and Mann–Whitney test for two-group comparisons. Pearson correlation analysis was used to determine the relationship between hs-CRP and echocardiographic parameters. Linear regression (Backward method) was performed to identify the association of hs-CRP with echocardiographic parameters. Correlation is significant at the p< 0.05.

## Results

In analysis of the data of the 50 patients with RHD who underwent PTMC after 36 months, mean transmitral valve gradient 36 months after PTMC (TMVG3) was 13 ± 3.9 (ranged 4–22), MVA (planimetry) was 2 ± 0.4 cm^2^ (ranged 1.2–3), MVA (PHT) was 1.9 ± 0.4 cm^2^ (ranged 1.2–3.1), PASP was 35 ± 7.4 mmHg (ranged 25 – 55), left atrial diameter was 46.4 ± 7.9 mm (ranged 33 – 67), and Wilkins score was 7.6 ± 0.8 (ranged 7–10), hs-CRP level was 2.7 ± 2.8 ngr/dl (ranged 0.1–10). Of these 41 patients were in sinus rhythm and 9 patients had atrial fibrillation (AF), 41 patients were New York Heart Association (NYHA) functional class I, 9 patients were class II. Table 1 shows distribution of mean TMVG before, immediate after and 36 months after PTMC that have been shown with TMVG1, TMVG2 and TMVG3 respectively and hs-CRP 36 months after PTMC. Tables 2 and 3 show association between hs-CRP and variables 36 months after procedure. Table 4 shows association between hs-CRP and demographic data 36 months after PTMC. No association was found between hs-CRP level and mean TMVG3, MVA by planimetry, PASP, MR grade, left atrial diameter, AF, Wilkins score, sex and age (p= 0.44, p= 0.18, p= 0.17, p= 0.39, p= 0.28, p= 0.38, p= 0.46, p= 0.12 and p= 0.73 respectively) in patients with CRMS who PTMC was done for them. Kruskal-Wallis test was used for association between hs-CRP and MR. 36 months after PTMC, 21 patients did not have MR, 21 patients had 1+ MR and 8 patients had 2+ MR. Mann-Whitney test was used for association between hs-CRP and NYHA functional class or AF. In linear regression (Backward method) MVA (planimetry and PHT), NYHA functional class and PASP were independent variables and hs-CRP was dependent variable. In linear regression analysis the relation between hs-CRP and MVA by PHT was not significant (Beta coefficient= 1.84, t= 2.00 and p= 0.051).

**Table 1:** Distribution of TMVG before, immediate after and 36 months after PTMC and hs-CRP serum level 36 months after procedure

**Variables**	**Mean ± SD**
TMVG1	23.77±7.1
TMVG2	2.7±6.1
TMVG3	13.0±3.9
Hs-CRP (ng/dl)	2.7±2.8

TMVG = Trans mitral valve gradient

**Table 2:** Correlation between hs-CRP and variables 36 months after procedure

**Variables**	**r**	p*
TMVG3	0.11	0.44
MVA (Planimetry)	0.19	0.18
MVA (PHT)	0.29	0.04
PASP	0.19	0.17
Left atrial diameter	0.15	0.28
Wilkins score	-0.10	0.46

MVA= Mitral valve area, PASP= Pulmonary arterial systolic pressure, PHT= Pressure half time method, TMVG= Trans mitral valve gradient, *= Pearson Correlation

**Table 3:** Correlation between hs-CRP, MR, NYHA functional class and AF; 36 months after procedure

**Variables**	**Minimum**	**Maximum **	**Mean±SD**	**N**	**p**
MR					0.39*
0	0.12	10	2.60±2.54	20	
1+	0.36	10	2.94±3.09	21	
2+	0.10	10	2.48±3.34	9	
NYHA functional class					0.04**
1	0.16	10	3.06±3.03	41	
2	0.10	3.54	1.19±1.22	9	
AF					0.38**
0	0.12	10	2.90±3.02	41	
1	0.10	5.57	1.89±1.99	9	

AF = Atrial fibrillation, MR = Mitral regurgitation, NYHA = New York Heart Association *= Kruskal-Wallis test, ** = Mann-Whitney test

**Table 4:** Correlation between hs-CRP and demographicData 36 months after PTMC

**Demographics**	**Age (Minimum)**	**Age (Maximum)**	**Mean±SD**	**N**	**p**	**r**
Sex					0.73*, 0.12**	0.05
Male	0.10	10	2.50±3.88	9		
Female	0.12	10	2.77±2.66	41		

*= Pearson Correlation, ** = Mann-Whitney test

## Discussion

In this cross-sectional study of patients with RHD who underwent PTMC, the hs-CRP serum levels were not correlated with MV restenosis. In other words, it was not correlated with mean TMVG, MVA, PASP, MR, Left atrial diameter, AF, and Wilkins score. Hs-CRP serum levels were not correlated with age and sex too. In our hypothesis inflammation or hs-CRP serum level, a marker of inflammation, was a cause of post PTMC restenosis, but in our linear regression analysis it was not significant. Many studies were performed about relationship between hs-CRP serum levels and MV stenosis before PTMC, but not investigated about relationship between hs-CRP serum levels and post PTMC restenosis. In a study by Seluck et al.,^15^ hs-CRP plasma levels in MS patients with AF was higher than in patients with Mitral Stenosis and in sinus rhythm. In addition, hs-CRP plasma levels in patients with Mitral Stenosis and in sinus rhythm did not differ from healthy controls and there was no association between plasma level of hs-CRP and MVA, mitral valve echocardiographic score and the MV gradient in either the sinus rhythm or AF groups. Furthermore, Chiu-Braga et al.^16^ found no association between inflammatory markers and the degree of MV stenosis. Other studies have shown that hs-CRP plasma levels increase in patients with RHD. In a hypothesis, so called antigenic mimicry, to explain the valvular damage in acute RF, is based upon an antigenic similarity between human heart valves and group A streptococci.^17^ It has been postulated that this autoimmune process leads to damage to the heart valves.^3^ In a study by Alyan et al.,^18^ hs-CRP plasma levels is positively correlated with mean Wilkins valve score value, PASP, presence of AF, left atrial diameter, left atrial area and presence of atrial spontaneous echo contrast (+) and inversely correlated with MVA. Similarity, Golbasi et al.^3^ have reported that hs-CRP is significantly higher in the chronic phase in patients with rheumatic valve disease than in patients with prosthetic valves and healthy subjects. Several studies^3,16,19^ have also demonstrated that CRP levels are increased in patients with CRMS and decreased after valve replacement. Our findings revealed that there was not significant inflammation several years after PTMC in patients with RHD. So it suggested that inhibition of inflammation after PTMC does not effect on long term outcome of patients. Several studies have revealed echocardiographic changes in patients with Mitral Stenosis after PTMC. PTMC is a safe and effective procedure in patients with Mitral Stenosis yielding satisfactory immediate results.^20^ Hasan-Ali et al.^21^ showed that PTMC produced significant morphologic and hemodynamic changes in the MVs. In a study by Saeki et al.,^22^ the PTMC immediate success rate, with considering MVA>1.5 cm^2^ or a MVA of more than twice the pre-PTMC valve and the absence of exacerbational MR of grade of 2 or more, was 95% in a middle-aged population. Also this study has shown that a large MVA after PTMC seems to correlate with a better capacity for daily activity in the later follow-up period. Echocardiographic characteristics of the mitral apparatus and the presence of AF before PTMC may be the significant predictors for the clinical outcomes. In a recent study by Devi et al.^23^ , restenosis after 10-year follow-up after successful PTMC, was influenced more by pre PTMC left atrial size, MVA, subvalvular fusion than on immediate post PTMC parameters. Former studies have shown that pre-PTMC Wilkins echocardiographic score and the presence of AF are reliable good predictors of restenosis.^24-27^

## Study limitations

The present study was limited by the small population, because the prevalence of RHD has decreased in Iran. Other major limitation of this study is that, it is cross-sectional study and CRP measurements was done, just at the end of the study, and not during the follow up period from the time of PTMC. We do not know about the variations in CRP during the follow up period that might be meaningful.

## Conclusion

In conclusion, our study has shown that there is no association between hs-CRP and MV restenosis in patients with RHD who underwent PTMC. Therefore, it has been postulated that the inflammation is not a cause of post PTMC restenosis.

## Ethical Issues

This study was approved by our local Ethics Committee.
